# Polymorphism of winter phenotype in Siberian hamster: consecutive litters do not differ in photoresponsiveness but prolonged acclimation to long photoperiod inhibits winter molt

**DOI:** 10.1186/s12983-021-00391-3

**Published:** 2021-03-18

**Authors:** Anna S. Przybylska-Piech, Michał S. Wojciechowski, Małgorzata Jefimow

**Affiliations:** 1 Department of Vertebrate Zoology and Ecology, Nicolaus Copernicus University, Toruń, Poland; 2 Department of Animal Physiology and Neurobiology, Nicolaus Copernicus University, Toruń, Poland

**Keywords:** Delayed life history effect, Winter phenotype, Polymorphism, Torpor, Molting, Metabolism

## Abstract

**Background:**

The theory of delayed life history effects assumes that phenotype of adult individual results from environmental conditions experienced at birth and as juvenile. In seasonal environments, being born late in the reproductive season affects timing of puberty, body condition, longevity, and fitness. We hypothesized that late-born individuals are more prone to respond to short photoperiod (SP) than early born ones. We used Siberian hamsters *Phodopus sungorus*, a model species characterized by high polymorphism of winter phenotype. We experimentally distinguished the effect of litter order (first or third) from the effect of exposure to long photoperiod (LP) before winter (3 months or 5 months) by manipulating the duration of LP acclimation in both litters. We predicted that, irrespective of the litter order, individuals exposed to long photoperiod for a short time have less time to gather energy resources and consequently are more prone to developing energy-conserving phenotypes. To assess effect of litter order, duration of acclimation to long days, and phenotype on basal cost of living we measured basal metabolic rate (BMR) of hamsters.

**Results:**

Individuals born in third litters had faster growth rates and were bigger than individuals from first litters, but these differences vanished before transfer to SP. Litter order or duration of LP acclimation had no effects on torpor use or seasonal body mass changes, but prolonged acclimation to LP inhibited winter molting both in first and third litters. Moreover, individuals that did not molt had significantly higher BMR in SP than those which molted to white fur. Although one phenotype usually predominated within a litter, littermates were often heterogeneous. We also found that over 10% of individuals presented late response to short photoperiod.

**Conclusions:**

Our data indicate that duration of postnatal exposure to LP may define propensity to photoresponsiveness, regardless of the litter in which animal was born. Existence of littermates presenting different phenotypes suggests a prudent reproductive strategy of investing into offspring of varied phenotypes, that might be favored depending on environmental conditions. This strategy could have evolved in response to living in stochastic environment.

## Background

The ability to respond to day length (photoperiodism) allows animals to change phenotype across the annual cycle. Response to shortening photoperiod consists of morphological, physiological and behavioral adjustments and results in development of winter phenotype. In winter, small mammals, which are mostly long-day breeders, regress gonads and cease reproduction [1–3], decrease body mass (*m*_b_) [4–6], some molt to white fur [7–10], and heterothermic species use torpor [11, 12]. These adjustments allow for energy savings and are considered beneficial for winter survival, yet individuals insensitive to changes in day length (nonresponding individuals), or individuals presenting only some of winter traits (partial-responding individuals) also exist in many populations [7, 8, 13–20]. The diversity of winter phenotypes may result from complexity of physiological and molecular mechanisms underlying photoresponsiveness [21, 22]. Melatonin, the hormonal signal of day length, enters multiple molecular pathways which control molting, torpor expression or gonadal regression [23, 24]. Although these pathways are often interrelated, winter traits may be regulated independently [23, 25–27].

According to the theory of delayed life history effects, phenotype of an adult individual results from environmental conditions experienced at birth, and later during growth and maturation [28–30]. Thus, it may be also affected by the time of birth during the reproductive season [31–33]. Late- and early born individuals differ in time it takes to reach puberty [34, 35], strategy of winter survival [33, 35], and longevity [36, 37]. Individuals born later during the reproductive season are often smaller [32, 38], grow slower [33] and have a lower probability of winter survival [36] than individuals born earlier. One can argue that late-born individuals have less time to grow and gather energy reserves before winter [32, 38].

In the Boreal and Temperate Zones seasonally changing day length correlates with changes in ambient temperature and resource availability. Day length experienced during development influences responsiveness to short day in adult Siberian hamsters *Phodopus sungorus*, a long-day breeder, which is a model animal in the studies of seasonal adjustments in physiology [16, 17, 39]. A majority of hamsters born and/or weaned under photoperiod shorter than 15 h respond to short days [17, 33], contrary to those born or weaned under 16 h photoperiod or longer [14, 15, 33]. Because photoperiod is related to time of the year, it has been proposed that nonresponsiveness to short days results from being born early in the reproductive season and exposure to long days during first weeks of life [17]. Butler et al. [33, 40] used simulated natural photoperiod to demonstrate that the proportion of nonresponding Siberian hamsters was greater in cohorts born under lengthening photoperiod (early born cohorts) than in cohorts born when days were shortening (late-born cohorts). In all cohorts responders always predominated, but even among hamsters born late in the season over 10% of individuals did not respond to short days [33]. This suggests that factors other than day length may play a role in development of the nonresponding phenotype.

Between April and September Siberian hamsters may deliver up to 5 or 6 litters [41], therefore photoperiod experienced by offspring at birth and during early development may considerably differ. Since being born late in the season is intrinsically related to shorter exposure to long photoperiod, it is hard to disentangle the effect of photoperiod and litter order on adult winter phenotype. In many mammalian species, both long- and short-day breeders, litters differ in offspring quality [42–45]. Depending on the species, subsequent litters can be bigger [44–46] or smaller [43, 47] or not different from each other [48, 49].

**Fig. 1:**
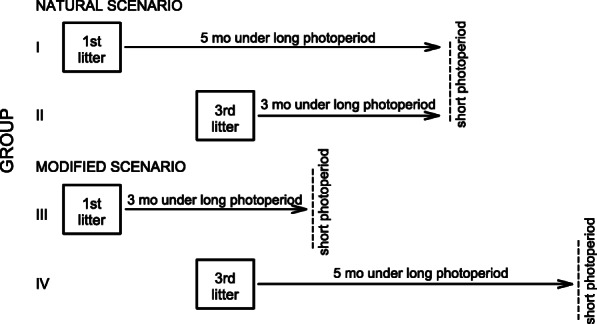
Experimental design. Siberian hamsters were divided into four groups differing in time of birth (1st or 3rd litters) and duration of acclimation to long photoperiod (3 or 5 months). See Methods for details

We proposed that extrinsic factors, such as access to energy resources prior to winter, influence the strategy of winter survival in small long-day breeding mammals and hypothesized that being born late in the reproductive season increases probability of subsequent development of photoresponsiveness. We aimed to experimentally distinguish the effect of litter order from the effect of exposure to long photoperiod (LP) before winter by manipulating the duration of LP acclimation in consecutive litters (Fig. 1). We predicted that the proportion of individuals developing traits characteristic for an energy-conserving phenotype (white fur, torpor use, and low *m*_b_) would be greater among individuals exposed to long photoperiod for a short time, irrespective of the litter in which they were born. As a model we used the Siberian hamster, photosensitive rodent that exhibits a high level of polymorphism of winter phenotype, from responding individuals, through individuals which develop only some of winter traits, to nonresponding ones [13, 50, 51]. Additionally, we compared basal metabolic rate (BMR) of animals from different experimental groups to assess effect of litter order, age, photoperiod and winter phenotype traits on basal energy consumption.

## Results

### Effect of litter order and duration of LP acclimation on offspring body mass

**Fig. 2:**
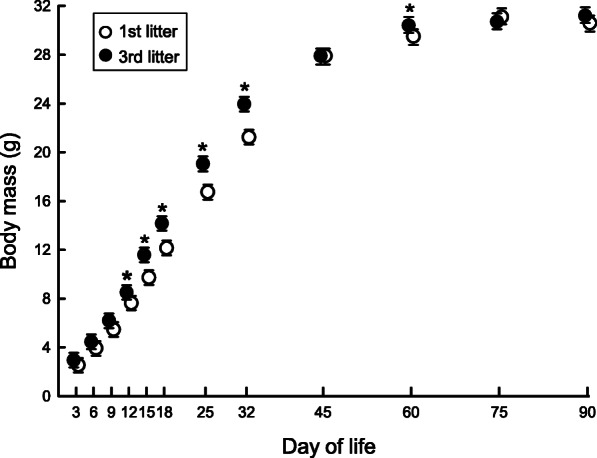
Body mass of offspring born in 1st (white symbols) and 3rd (black symbols) litters. Dots indicate estimated marginal means, and whiskers are 95% confidence intervals. Asterisks show days when litters differed significantly

Offspring *m*_b_ increased with dam *m*_b_ (LME: F (1, 23) = 5.951, *P* = 0.026) and was negatively correlated with litter size, but the latter was true only in first litters (litter order × litter size, LME: F(1, 372) = 5.978, *P* = 0.015). Offspring had similar *m*_b_ for the first nine days of life, irrespective of litter order, but thereafter offspring from first litters were smaller than third litter individuals (litter order × age, LME: F(1, 351) = 5.861, *P* <  0.001; Fig. 2). Although differences in body mass between consecutive litters ranged from 9.56% at day 12 of life to 16.43% at day 15, it vanished between day 45 and day 90. However 60-day old hamsters from third litters were bigger than individuals from first litters (litter order × age, LME: F(1, 600) = 4.785, *P* = 0.003, Fig. 2). Between days 45 and 90 males from first and third litters did not differ but females born in first litter were smaller than those born in third litter (litter order × sex, LME: F(1, 485) = 6.581, *P* = 0.011). Body mass of offspring acclimated to LP for 3 or 5 months did not differ prior to transfer to SP (GLM: F(1, 199) = 1.608, *P* = 0.206). Model designs and results of the analysis of variance are given in Table 1, whereas data on hamster *m*_b_ are given in Table 2.

**Table 1:** Results of the type III analysis of variance calculated for offspring body mass in Siberian hamster

Model no	Trait	Model	Factors	F(df)	*P-value*
1	*m*_b_ until 32-day of life	LME	**age**	**633.54 (7, 55)**	**0.006**
			litter order	3.49 (1, 374)	0.062
			**litter size**	**9.45 (2, 283)**	**0.002**
			**dam** ***m***_**b**_	**5.95 (1, 23)**	**0.026**
			**age** × **litter order**	**5.86 (7, 351)**	**< 0.001**
			**litter order**×**litter size**	**5.98 (1, 372)**	**0.015**
2	*m*_b_ between 45 and 90-day of life	LME	**age**	**108.05 (3, 601)**	**< 0.001**
			litter order	0.58 (1, 188)	0.447
			**sex**	**318.83 (1, 485)**	**< 0.001**
			**age × litter order**	**4.78 (3, 601)**	**0.003**
			**litter order × sex**	**3.58 (1, 485)**	**0.011**
3	*m*_b_ prior to transfer to SP	GLM	litter order	2.44 (1, 199)	0.120
			duration of LP acclimation	1.61 (1,199)	0.206
			**sex**	**136.26 (1, 199)**	**<0.001**
4	*m*_b_ after 16 weeks under SP	GLM	litter order	0.81 (1, 186)	0.368
			duration of LP acclimation	1.55 (1, 186)	0.214
			sex	0.34 (1, 186)	0.563
			**torpor use**	**17.02 (2, 186)**	**< 0.001**
			**molting**	**81.71 (2, 186)**	**< 0.001**
			**initial mb**	**11.10 (1, 186)**	**0.001**
			**sex × torpor use**	**4.58 (2, 186)**	**0.034**
			**sex × molt**	**9.45 (2, 186)**	**0.002**

*m*_*b*_ body mass, *SP* short photoperiod, *LP* long photoperiod, *LME* linear mixed effect model, *GLM* general linear model. Significant effects are indicated in bold

**Table 2:** Estimated marginal means ± SE for body mass of offspring from first and third litters, calculated from models one and two (Table 1.), compared pairwise with Tukey’s HSD test adjusted for multiple comparisons. Significant differences are indicated in bold

Day of life	Body mass (g)		Post-hoc *P -value*
	1^st^ litter	3^rd^ litter	
3	2.54 ± 0.30	2.95 ± 0.30	0.274
6	3.91 ± 0.30	4.45 ± 0.30	0.159
9	5.46 ± 0.30	6.19 ± 0.30	0.055
12	7.63 ± 0.30	8.50 ± 0.29	**0.021**
15	9.72 ± 0.30	11.57 ± 0.30	**0.001**
18	12.14 ± 0.30	14.16 ± 0.30	**0.001**
25	16.74 ± 0.31	19.05 ± 0.31	**0.001**
32	21.24 ± 0.30	23.95 ± 0.31	**0.001**
45	27.90 ± 0.33	27.90 ± 0.33	0.998
60	29.50 ± 0.33	30.40 ± 0.33	**0.042**
75	31.10 ± 0.33	30.70 ± 0.33	0.437
90	30.60 ± 0.33	31.20 ± 0.33	0.174

Significant differences are indicated in bold

### Parental effect on offspring phenotype

None of the parental pairs delivered offspring which developed only one phenotype (the same set of winter traits) in response to short photoperiod. In some pairs, most offspring turned white and used torpor, whereas in other pairs grey offspring that did not use torpor predominated. Different phenotypes among littermates were more common in first (in 24 out of 25 parental pairs) than in third litters (in 17 out of 25 parental pairs) (χ^2^(1, 50) = 6.640, *P* = 0.010). For example, in three parental pairs littermates showed all possible combinations of winter traits, and therefore phenotypes: white fur and torpor use, white fur without torpor use, grey fur and torpor use, and grey fur without torpor use. In another 14 pairs, offspring presented three different phenotypes and in 8 pairs only two different combinations of traits. Because we did not know parental phenotype, we were not able to calculate heritability of winter traits. However, the goodness of fit of models with and without random effect of parental ID differed significantly (*P* = 0.001), suggesting a strong parental effect on offspring phenotype.

### Effect of litter order and duration of LP acclimation on photoresponsiveness

During the first 16 weeks of acclimation to SP, 94 out of 200 animals used daily torpor and 125 animals molted to white fur. Between weeks 20 and 37 of acclimation to SP, another 23 animals molted and 29 entered torpor for the first time. Generally, experimental groups did not differ in propensity to use torpor (litter order × duration of LP acclimation χ^2^ (1, 200) <  0.001, *P* = 0.993) or molting (litter order × duration of LP acclimation χ^2^(1, 200) <  0.001, *P* = 0.988). Neither litter order (χ^2^(2, 200) = 0.194, *P* = 0.907) nor duration of LP acclimation (χ^2^(2, 200) = 1.660, *P* = 0.436; Table 3) affected the use of torpor. There was also no effect of litter order on molting (χ^2^(2, 200) = 0.128, *P* = 0.938; Table 3). However, in groups acclimated to LP for 3 months we observed 71% individuals which molted to white in response to SP, and only 54% in groups acclimated to LP for 5 months (χ^2^(2, 200) = 6.36, *P* = 0.041; Table 3). In the latter groups, we observed also almost two times more late-responding individuals than in groups acclimated to LP for 3 months (Table 3).

**Table 3:** Effect of litter order and duration of long photoperiod (LP) acclimation on torpor use and molting in Siberian hamsters born in first or third litters, and acclimated to LP for 3 or 5 months. Each group consisted of 50 individuals acclimated to the short day for 16 weeks. Bold text indicates statistically significant differences (*P* < 0.05) between groups acclimated to LP for 3 or 5 months

		Torpor use			Molting		
		Number of individuals			Number of individuals		
Duration of LP acclimation (months)	Litter	using torpor	not using torpor	using torpor after 20 weeks under SP	molting to white	remaining grey	molting to white after 20 weeks in SP
3	1	24	20	6	**34**	12	**4**
	3	27	17	6	**37**	9	**4**
5	1	22	20	8	**28**	15	**7**
	3	21	20	9	**26**	16	**8**

Each group consisted of 50 individuals acclimated to the short day for 16 weeks

Values indicated in bold show significant differences (*P* < 0.05) between groups acclimated to LP for 3 or 5 months

The higher the initial *m*_b_ of hamsters at the end of acclimation to long days, the greater the decrease of *m*_b_ after acclimation to short days (GLM: F(1, 198) = 14.82, *P* <  0.001). Neither litter order (GLM: F(1, 186) = 1.287, *P* = 0.258) nor duration of LP acclimation (GLM: F(1, 186) = 1.345, *P* = 0.248) affected *m*_b_ changes after 16 weeks in SP. We found that *m*_b_ change correlated with other winter phenotype traits. Namely, individuals using torpor (regardless of the time spent in SP) lost between 1 and 9% of initial *m*_b_ while individuals that did not use torpor maintained initial *m*_b_ or even gained it to 3% (GLM: F(1, 186) = 11.134, *P* <  0.001). Changes of *m*_b_ in different molting categories were related to sex (sex × molting GLM: F(1, 186) = 5.721, *P* = 0.004). Both, grey males and grey females gained up to 4.5% of initial *m*_b_, whereas white males and white females lost between 9 and 14% of initial *m*_b_. Within animals that molted after 20 weeks under SP, males gained around 5%, and females lost over 5% of initial *m*_b_.

**Fig. 3:**
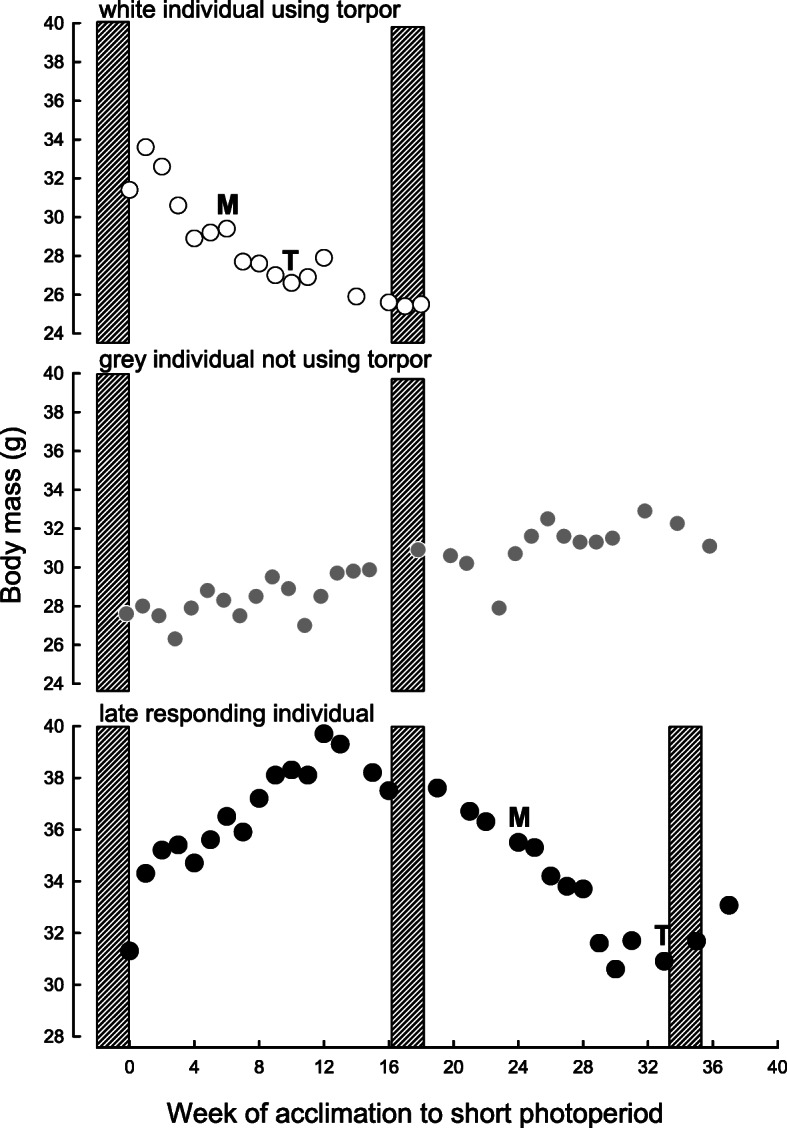
Changes in body mass in representative white individual using torpor (top panel), grey individual not using torpor (middle panel) and individual using torpor and molt to white fur later than after 20 weeks under short photoperiod (SP) (bottom panel). White individuals using torpor were transferred to LP after BMR measurements in SP, and there are no data points afterward. Hatched bars indicate time of BMR measurements. M – stage 3 of molting; T – first episode of torpor

Changes of *m*_b_ during further acclimation to SP in late responding animals correlated with their phenotype. Animals that molted within 16 weeks in SP and started to use torpor later than 20 weeks in SP did not change their *m*_b_ any further. Most individuals that both molted and started to use torpor later than after 20 weeks in SP decreased their *m*_b_ by approximately 16.3 ± 10.2% (Fig. 3) but some of them also gained *m*_b_ by 10.1 ± 7.6%. The two grey individuals that started to use torpor later than after 20 weeks in SP differed between each other, one lost *m*_b_ while the other maintained constant *m*_b_.

### Basal metabolic rate

Basal metabolic rate was repeatable in 3-month-old animals (τ = 0.263, *P* = 0.001) and between acclimation to long photoperiod and short photoperiod (τ = 0.235, *P* = 0.001), but not in late responders between subsequent measurements (τ = 0.060, *P* = 0.151).

**Fig. 4:**
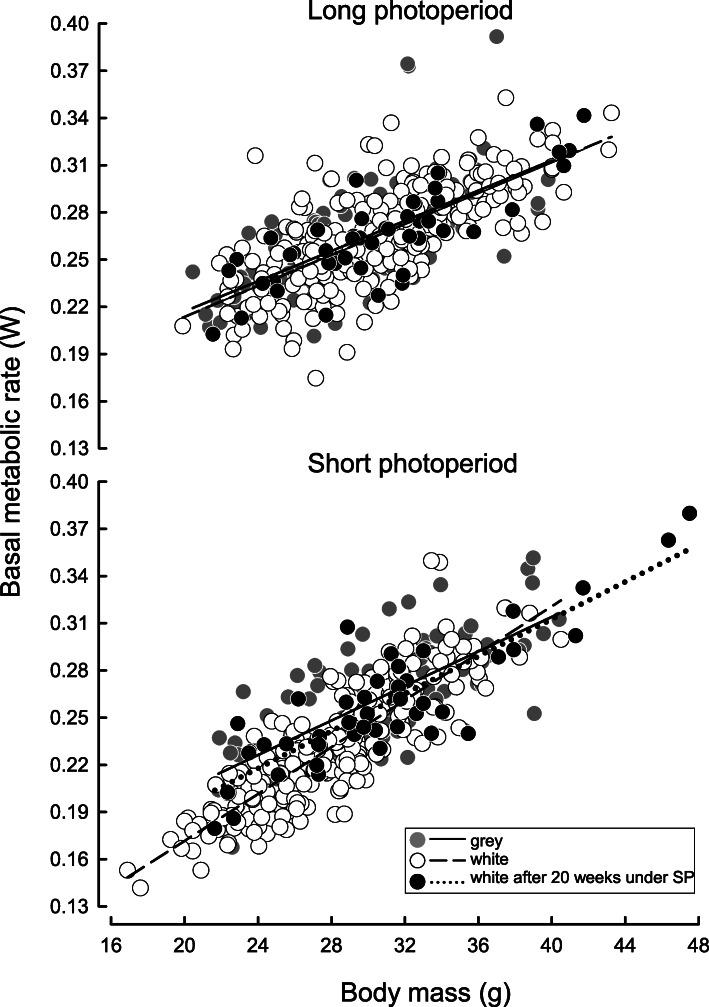
Relationship between basal metabolic rate (BMR) and body mass in white and grey individuals and in individuals that molted to white after 20 weeks under short photoperiod. Top panel shows data recorded in long photoperiod prior transfer to short photoperiod, and bottom panel shows data recorded after 16 weeks in short photoperiod. Regression lines did not differ between groups in long photoperiod but did differ in short photoperiod (see text). BMR was measured at ~28.5 °C

Hamsters born in first or third litters did not differ in BMR at the age of 3 months (LMM: F(1, 217) = 1.486, *P* = 0.224) but after adjusting for *m*_b_ females had higher BMR than males (0.273 ± 0.002 W and 0.261 ± 0.002 W respectively; LMM: F(1.224) = 15.218, *P* <  0.001). Basal metabolic rate decreased from 0.261 ± 0.004 W in long photoperiod to 0.247 ± 0.004 W after 16 weeks of acclimation to short photoperiod (LMM: F(1.591) = 66.739, *P* <  0.001, Fig. 4). This decrease depended on molting category, litter order and duration of LP acclimation. Namely, individuals acclimated to long days for 3 months did not change BMR between photoperiods, whereas those acclimated to LP for 5 months decreased BMR after being transferred to short days (photoperiod × duration of LP acclimation LMM: F(1, 595) = 44.234, *P* <  0.001). While all individuals had similar BMR in long photoperiod, after 16 weeks under short photoperiod BMR did not differ only among hamsters from third litters. Among hamsters from first litters, grey individuals had 6–10% higher BMR than white ones, and also than individuals that molted to white fur later (photoperiod × litter order × molting; LMM: F(1, 590) = 5.376, *P* = 0.005, Fig. 4). In late responding first litter individuals BMR increased in the course of acclimation to short photoperiod, whereas in individuals from third litters BMR was the same between measurements in short photoperiod (litter order × time of measurement LMM: F(1, 139) = 3.615, *P* = 0.029). Model designs and results of the analysis of variance are given in Table 4.

**Table 4:** Results of the type III analysis of variance calculated for basal metabolic rate (BMR) in Siberian hamster

Model no	Trait	Model	Factors	F(df)	*P-value*
1	BMR in 3 months old animals	LME	***m***_**b**_	**189.79 (1, 222)**	**<0.001**
			litter order	1.49 (1, 208)	0.224
			**sex**	**15.22 (1, 224)**	**<0.001**
			*m*_b_×litter order	2.87 (1, 209)	0.091
2	BMR after16 weeksunder SP	LME	***m***_**b**_	**715.89 (1, 412)**	**< 0.001**
			litter order	0.10 (1, 191)	0.752
			duration of LP acclimation	0.37 (1, 190)	0.542
			**sex**	**24.43 (1, 274)**	**< 0.001**
			**photoperiod**	**66.74 (1, 591)**	**< 0.001**
			**molting**	**9.07 (2, 190)**	**0.001**
			**duration of LP acclimation × molting**	**3.31 (2, 189)**	**0.038**
			duration of LP acclimation × photoperiod	0.18 (1, 595)	0.673
			**litter order × molting**	**4.61 (2, 190)**	**0.011**
			litter order × photoperiod	2.92 (1, 590)	0.087
			photoperiod × molting	1.38 (2, 594)	0.252
			duration of LP acclimation × photoperiod × molting	0.35 (2, 591)	0.707
			**litter order × photoperiod×molting**	**5.38 (2, 590)**	**0.005**
3	BMR of late responding individuals	LME	***m***_**b**_	**204.38 (1, 40)**	**<0.001**
			litter order	0.02 (1, 99)	0.877
			duration of LP acclimation	2.78 (1, 22)	0.109
			**sex**	**7.67 (1, 24)**	**0.011**
			**time of measurement**	**6.23 (2, 137)**	**0.002**
			molting	0.01 (1, 22)	0.940
			**litter order × time of measurement**	**3.62 (2, 139)**	**0.029**

*m*_*b*_ body mass, *SP* short photoperiod, *LP* long photoperiod, *LME* linear mixed effect model. Significant effects are indicated in bold

## Discussion

Early life events may profoundly affect adult phenotype [31–33]. We found that individuals that spent 3 months under long photoperiod were more prone to molt to a white fur than individuals from groups acclimated to long photoperiod for 5 months (Table 3). Additionally, white individuals had lower basal energy expenditure in short photoperiod than grey ones (Fig. 4). This supports our prediction that individuals exposed to long photoperiod for a short time, irrespective of the litter in which they were born, were more prone to develop an energy-conserving phenotype. However, our prediction that individuals born later during reproductive season have less time to gather energy reserves before winter was not supported because initial differences in body mass between hamsters originating from consecutive litters vanished before transfer to short photoperiod (Fig. 2). We also found that litter order or duration of LP acclimation had no effect on torpor use or *m*_b_ change.

### Delayed life history effect and offspring body mass

In the present study, consecutive litters differed in growth rate and individuals born in third litters grew faster than individuals born in first litters (Fig. 2, Table 2). In many mammalian species, including Siberian hamsters, litter order or time of birth during reproductive season affect litter or offspring quality [42–44, 46, 47]. It is true both for long- and short-day breeders. Long-day breeders, which are mostly small mammals with short gestation periods, mate in spring, while short-day breeders, like large ungulates, mate in autumn and have long gestation period. Although these groups differ in gestation lengths, parturition takes place at the time of year with highest resource availability. Therefore, late birth during the reproductive season may affect quality of mammalian offspring. Offspring of short-day breeder, red deer *Cervus elaphus*, born late during reproductive season were smaller than early born ones [36]. In European rabbits *Oryctolagus cuniculus* offspring from subsequent litters [47] were smaller than offspring born in earlier litters, whereas in garden dormice *Eliomys quercinus* [52], Uinta ground squirrels *Spermophilus armatus* [46], or Siberian hamsters [44] this trend was opposite. Differences in *m*_b_ between consecutive litters might have resulted from the efficiency of parental care, dam’s age, and body condition. In European rabbits previous reproductive activity might have had negative effect on current reproduction since first litters had higher growth rate than consecutive ones [47]. Conversely, laboratory mice [53] and rats [54] increased lactation performance and therefore reproductive effectiveness with successive litters. However, increase in reproductive effectiveness was limited to first two or three litters, and then it constantly decreased [55, 56]. Quality of offspring might be related also to maternal age and body condition [55, 57]. Bigger dams of both long- and short-day breeders delivered bigger offspring [58–60], but reproductive effectiveness depended also on time of the year when the dam was born [55].

In a previous study we found that offspring *m*_b_ in Siberian hamsters was related to parental phenotype [44] and nonresponding hamsters delivered bigger offspring than responding ones. Since responding individuals regress gonads, energy allocated into gonadal recrudescence and rebuilding of body reserves may reduce resources available for reproduction. Presumably this is why photoresponding hamsters commenced breeding later than nonresponding ones [44]. Regardless of parental phenotype offspring from successive litters were always bigger than earlier ones ([44], present study). In the present study, a negative relationship between litter size and offspring *m*_b_ only in first litters may suggest that after winter all individuals, regardless of their photoresponsiveness and gonadal status, might be in negative energy balance. It clearly indicates that reproduction-related trade-offs appear only when energy resources are limited.

We predicted that individuals born later in the reproductive season would develop traits of an energy-conserving phenotype, because these individuals have less time for gathering energy reserves, and they are considered to have lower chance to survive and reproduce next year [34, 38, 61]. On the one hand faster growth rate of offspring from subsequent litters could have compensated for late birth [46, 62, 63]. On the other hand, compensatory growth might have delayed consequences, such as inhibited growth in adults [64–66]. Since differences in *m*_b_ between litters vanished already at day 45 of life (Fig. 2), the latter explanation may be the case in Siberian hamsters. Under natural conditions, this species breeds from April to September [41]. Even if dams give birth every 23–30 days [67], third litters are born in June or July, i.e. 2–3 months before winter. Since we did not find differences between litters in body mass or BMR at the age of 3 months and prior to transfer to SP, it suggests that this time is long enough to complete growth before winter.

### Delayed life history effect and photoresponsiveness

We observed the whole spectrum of possible winter phenotypes among the study animals, including late responders that developed winter traits after more than 20 weeks under SP. It was previously reported that photoresponsiveness diminished with animal age and vanished after first year of life [6, 8]. However, since hamsters from first and third litters were born ~ 60 days apart, age cannot explain differences in photoresponsiveness.

Being born late in reproductive season decreased probability of winter survival in hibernating rodents [46, 68]. To our knowledge, direct effects of the duration of exposure to long photoperiod on physiology of seasonal rodents originating from early and late litters have not been tested before. Butler et al. [33, 40] found that more nonresponding hamsters were born in lengthening photoperiod (early cohorts), but nonresponding individuals were still present among individuals born under shortening photoperiod (late cohorts) [33, 40]. Nevertheless, the study did not report whether hamsters originated from first or consecutive litters.

Prolonged acclimation to LP reduced the number of individuals that molted to white fur in response to SP (Table 3). Seasonal molting is related to decreased prolactin level [21, 22]. Since Siberian hamsters born early during reproductive season may reach puberty before their first winter, prolonged acclimation to long photoperiod may increase prolactin level and therefore inhibit molting. Lack of molting can be regarded as disadvantageous because white fur increases insulation [69], but Boratyński et al. [51] did not find differences in thermal conductance between winter and summer acclimated Siberian hamsters. Although energy savings due to molting in small mammals are considered to be limited [9, 70], they may still be present. Here we found that after acclimation to short photoperiod, BMR of white animals was 10% lower than BMR of grey individuals (Fig. 4). Since high prolactin level has been shown to increase food intake, adipogenesis, and *m*_b_ [71, 72] it may also explain high BMR of grey individuals acclimated to short days.

Neither litter order nor duration of LP acclimation affected torpor use or *m*_b_ change. As far as we know, this is first study which experimentally tested the effect of these factors on torpor use in Siberian hamsters. Torpor is an effective way to reduce energy expenditure under SP [73–75] and propensity for torpor increased in cold or after fasting [76, 77]. It is plausible that we did not observe any effect of litter order and duration of LP acclimation on torpor use because of constant and relatively high ambient temperature during acclimation and food available ad libitum. This might also be a reason for similar *m*_b_ prior to transferring animals to SP and for similar time courses of *m*_b_ changes under SP in all groups. Although, individual body condition and energy reserves did not affect photoresponsiveness under mild conditions, it may be of great importance in harsh environments.

In the present study, prolonged acclimation to long days increased the number of late responding hamsters (Table 3). In these individuals the sequence of seasonal changes was the same as in typically responding ones (response after 12–16 weeks under SP). Namely, decrease in *m*_b_ was followed by molting and finally, occurrence of torpor (Fig. 3). However, late responders were in their winter phenotype for shorter time because photorefractoriness occurred at a similar time as during the typical response to SP [11, 78]. Although the phenomenon of late responders is known [11, 78], the mechanism of delayed response to winter remains unexplained. It does not seem to be a laboratory artefact, because both abrupt and gradual changes from long to short days led to development of winter phenotypes and any differences vanished before the 12th week of SP acclimation [16, 79].

At first glance, existence of late responders under natural conditions seems to be difficult to understand. However, this picture may change when we account for the fact that other winter-related phenomena, such as snowfall or low ambient temperature, may be shifted towards spring. Since late responders from third litters were able to maintain low BMR for the entire course of acclimation to short photoperiod, their energy reserves might have been maintained for a longer time. Therefore, prolonged cold or unexpected snowfall in spring may favor a late winter response and explain maintenance of late responders in a population.

### Polymorphism in winter traits within a litter

**Fig. 5:**
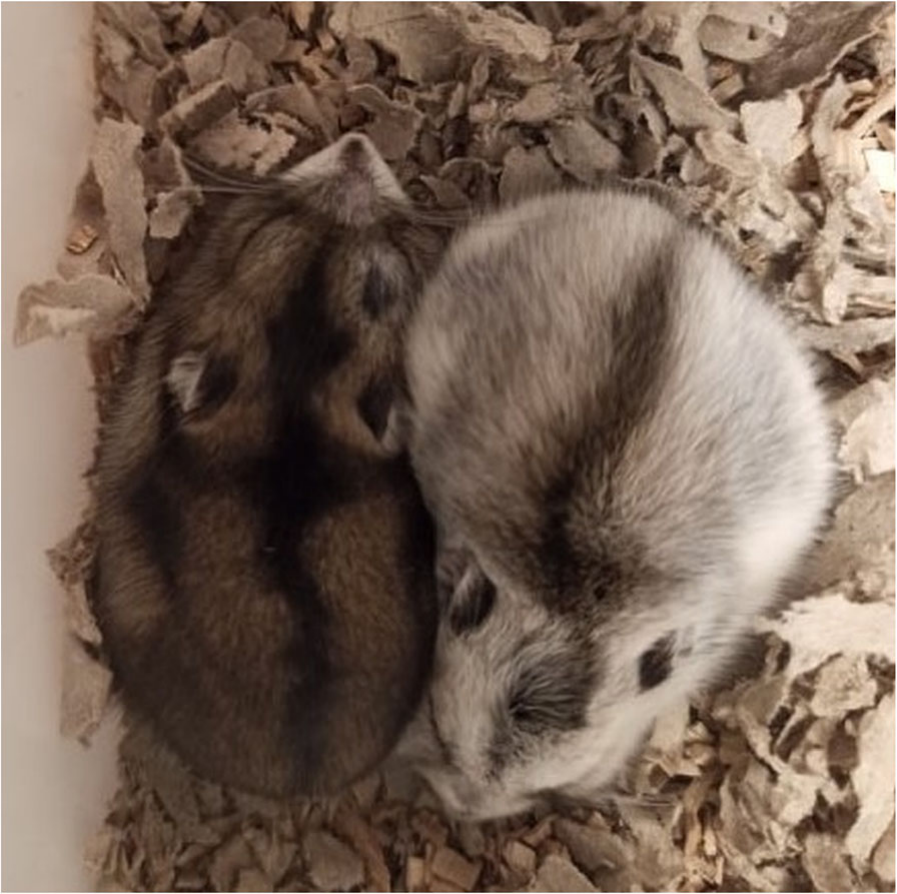
Littermates of Siberian hamsters presenting different winter phenotypes. Left one remained grey and never used daily torpor, whereas right one molted to white and used daily torpor. Picture was taken after 14 weeks under short photoperiod (8L:16D)

In the present study none of the parental pairs delivered offspring of one phenotype. Within a litter there were individuals of different responsiveness to SP, but in a few parental pairs one offspring phenotype predominated, suggesting heritability of the winter phenotype. Previous reports showed that phenotype was heritable in Siberian hamsters and white-footed mice [3, 14, 80] and artificial selection led to increase of photoresponsiveness [81]. Since our colony is outbred, parental pairs were paired randomly and we did not control for their winter phenotype, calculation of heritability of winter traits was impossible. Polymorphism was more common in the first litters suggesting that individuals born earlier during the reproductive season may show higher variability in the response to shortening days, than less flexible, bigger and faster growing individuals from third litters. In our breeding colony some littermates that were maintained together in the same cage and under the same conditions developed different winter phenotypes (Fig. 5). According to Balanced Polyphenism Hypothesis [82, 83] offspring of different phenotypes may increase parental fitness, because each phenotype can be advantageous under specific environment conditions.

## Conclusions

Winter phenotype may be considered as a suite of morphological, physiological and behavioral adjustments, but it seems that particular winter traits develop independently. To the best of our knowledge this is the first time when existence of different phenotypes among littermates is reported. Goldman and Goldman [15] proposed that all hamsters are born as responsive to short photoperiod but in some individuals this response is lost due to extrinsic factors (e.g. long photoperiod during postnatal development). Indeed, time of birth during the season may affect strategy of winter survival since prolonged acclimation to long days inhibited seasonal molting and increased energy expenditure. Individuals which do not respond to SP and have higher BMR may have a lower chance to survive harsh winter than individuals which responded to SP with all physiological and morphological traits. Conversely, nonresponding individuals may develop a late response which may be favored under particular environmental condition. Although we did not find any delayed life history effects on torpor use or seasonal *m*_b_ changes, we suggest that under harsh environmental conditions and when resources are limited, or in younger individuals, energy supplies gathered before winter have significant effect on development of these winter traits and overall photoresponsiveness.

## Methods

### Animals

All experimental procedures were approved by the Local Committee for Ethics in Animal Research in Bydgoszcz, Poland (decisions: 8/2018, 22/2018, 48/2018, 18/2019). We used 200 Siberian hamsters, 100 males and 100 females, originating from our outbred colony maintained at the Faculty of Biological and Veterinary Sciences at the Nicolaus Copernicus University in Toruń, Poland. Half of animals were born in the first litters and another half in the third litters. All individuals were born between late May and late August. Litters were weaned at day 18 and each litter shared one cage until day 32. After that, we randomly selected 2 males and 2 females from each litter. Siblings of each sex were maintained together until they were two-months old. Thereafter each hamster was assigned to experimental group and transferred to an individual cage in which it was maintained throughout entire experiment. Groups did not differ in body mass at the age of 2 months (UNIANOVA F(1, 200) = 1.126, *P* = 0.340). To estimate growth rate, whole litters were weighed every 3 days until weaning and then every week until day 32. Weighing was continued every two weeks until transfer to short photoperiod. After birth hamsters were maintained under long photoperiod (LP; 16 L:8D, lights on at 04:30 a.m.) at ambient temperature of 20 ± 2 °C. The 16 h day does not inhibit further response to short photoperiod [14, 17]. All animals were maintained in standard laboratory cages (Tecniplast, 1245, Italy, 33 × 20 × 18 cm) with deciduous wood chips as bedding material. Hamsters were fed with standard rodent food with higher content of protein and fat (60% of carbohydrates, 10% of fat and 30% of protein; Labofeed H standard, Morawski, Kcynia, Poland) until the second month of life, and afterwards with standard maintenance food (67% of carbohydrates, 8% of fat and 25% of protein; Labofeed B standard, Morawski, Kcynia, Poland). Animals were supplied with drinking water ad libitum.

### Experimental protocol

To determine the effect of time of birth on offspring photoresponsiveness, we set four experimental groups of 50 individuals each, which differed in the time of birth or in the duration of acclimation to long photoperiod after birth (Fig. 1). Although Siberian hamsters may breed immediately after parturition of previous litter [41, 84], a post-implantation embryonic diapause is common [67] and females usually deliver consecutive litters every ~ 23–30 days [44]. In our study third litters were ~ 2 months (50–65 days) younger than the first litters. Hamsters originating from first and third litters were randomly assigned to experimental groups which differed in duration of post-natal acclimation to long photoperiod. Two groups were intended to imitate natural conditions (Natural Scenario in Fig. 1). Group I was composed of animals born in first litters that were acclimated to LP for ~ 5 months and then transferred to short photoperiod (SP; 8 L:16D, lights on at 08:30 a.m.). Hamsters from Group II were born in third litters and since they were transferred to SP together with the first group, they were acclimated to LP for ~ 3 months. Groups III and IV were used to control for the effects of litter order and duration of LP acclimation on the development of winter traits (Modified Scenario in Fig. 1). Individuals from group III were born in the first litters, but their acclimation to LP was shortened to 3 months. In group IV, LP acclimation of animals born in the third litters was lengthened to ~ 5 months. To test our hypothesis, we used abrupt change of photoperiod, which allowed to precisely measure time spent in both photoperiodic regimes. Despite limitations discussed by Gorman et al. [16], this protocol has been widely used to induce a seasonal response in Siberian hamsters [7, 14, 85–89]. It has also been reported that proportion of nonresponding individuals was not related to the method of photoperiod transition from summer to winter [16]. To account for parental effect, all offspring were derived from 25 parental pairs. Each parental pair was represented by one male and one female offspring in each group, resulting in 8 offspring per parental pair across all groups.

### Determination of winter traits

After transfer to SP, hamsters were maintained individually, in the same type of cages and at the same ambient temperature as in LP. Development of winter traits was determined after 16 weeks under SP based on pelage color, torpor use and *m*_b_ change. Traditionally, individuals were classified as responders, nonresponders, or partial responders based on set of several winter traits, such as daily torpor and pelage color or gonadal regression and *m*_b_ loss [8, 13, 40, 90]. However, because winter traits are controlled by different hormonal and molecular pathways [21, 22] and they may develop independently in response to SP, such simple classifications become debatable. Here, we analyzed effect of delayed history effects on each winter trait separately. An individual was classified as using torpor if it entered at least one torpor episode (subcutaneous temperature ≤ 32 °C, stereotypical posture, reduced responsiveness). To determine this, hamsters were injected subcutaneously in interscapular region with thermosensitive passive integrated transponders (BioTherm 13, Biomark, Boise, ID, USA) after 2 to 4 weeks of SP acclimation. Although the manufacturer-reported temperature range of transponders is 33 °C to 43 °C, we calibrated them in a water bath against a high-precision mercury-in-glass thermometer between 27.0 °C and 40.0 °C, which allowed us to measure subcutaneous temperature (*T*_sc_) lower than 30 °C. Any *T*_sc_ lower than 24 °C was indicated on the reader as “Low”. These transponders allowed for remote monitoring of *T*_sc_ and torpor use during acclimation. Daily controls of hamster *T*_sc_ began four weeks after implantation. Between 10:30 and 14:30 (2 to 4 h after lights on), at a random time, we read *T*_sc_ with a remote reader (HPR plus, Biomark, Boise, ID, USA) and additionally noted if animal were in characteristic torpid ball-shape posture and whether they responded to gentle opening of the cage. We classified individuals as white, if we observed at least stage 3 of winter fur according to Figala scale (where 1 is dark grey with black stripe on a back and 6 is white without a stripe [91]). We did not score the intensity of fur color change any further. To assess *m*_b_ changes under SP, hamsters were weighed (to the nearest 0.1 g; Scout Pro 200, Ohaus, USA) every week during first 16 weeks of acclimation to SP and then every one or two weeks until week 40. In some individuals, so-called “late responders”, winter traits may appear even after 32–37 weeks under SP [11]. Hence, all hamsters that did not develop either white fur or torpor use after 16 weeks were maintained under SP up to ~ 40 weeks.

### Measurement of basal metabolic rate

Basal metabolic rate was measured in normothermic hamsters at the age of three months. In groups I and IV (Fig. 1), which were acclimated to LP for 5 months, BMR was additionally measured at the age of five months, just before transfer to SP. Then, BMR was measured in all animals again after 16 weeks under SP. In late responders BMR was measured only if we observed torpor use for the first time after 20 weeks in SP and this measurement was done as soon as torpor was observed. Measurements were always done in a repetition, 7 days apart, to assess repeatability of BMR. Animals were weighed before and after each metabolic measurement.

Basal metabolic rate was measured by indirect calorimetry using an open-flow respirometry system (Sable Systems International, Las Vegas NV, USA; henceforth: SSI), as described in Jefimow et al. [92]. Measurements were done in the thermoneutral zone of Siberian hamster (*T*_a_ ~ 28.5 °C; [93]) and gas exchange was measured for ~ 7 h which is long enough to ensure post-absorptive state in this species [93]. BMR was calculated as a rate of the most stable 2 min of O_2_ consumption during last three hours of the test. Animals were sealed in 0.85 L chambers made of polypropylene food containers (HPL 808, Lock&Lock, Hana Cobi, South Korea) which were placed in a temperature-controlled cabinet (ST-1200 BASIC, Pol-Eko-Aparatura, Wodzisław Śląski, Poland). We measured respiratory gas exchange of 14 animals simultaneously, using two parallel respirometry systems. Air was pulled from outside the building using an air pump (DOA-P501-BN, Gast Manufacturing INC., Michigan, USA) and dried in columns of silica gel. The main air stream was split into chambers and a reference gas stream. We regulated flow rate at ~ 500 ml min^− 1^ upstream of each respirometry chamber using a precise needle valve. After air left chambers, a computer-controlled multiplexer (Intelligent Multiplexer V3, SSI) sequentially selected gases from each chamber. Gas exchange of each animal was measured for 5 min every 44 min with reference gas concentration readings every 20 min. Air flow was measured downstream of animal chambers using mass flow meter (FlowBar-8, SSI). Then the air stream was subsampled at ∼200 ml min^− 1^ and water vapor pressure of the subsampled air was measured with a water vapor analyzer (RH-300, SSI). Air was then dried using a nafion dryer tube (product number 17049, VacuMed, Ventura, CA, USA) embedded in silica gel, and a column of magnesium perchlorate (product number 11636.36, VWR International, Gdańsk, Poland). Subsequently, concentrations of CO_2_ and O_2_ were measured. In one system, we measured both rates of O_2_ consumption (*V̇O*_*2*_) and CO_2_ production (*V̇CO*_*2*_). In the second system, only *V̇O*_*2*_ was measured. We used O_2_ (FC-10a, SSI) and CO_2_ (CA-10, SSI) analyzers. All elements of the respirometry system were connected to PC via an analog-to-digital interface (UI2, SSI). Data were acquired using ExpeData software (SSI) at 0.5 Hz.

Metabolic rate (MR, *W*) was calculated using oxyjoule equivalent calculated after Lighton et al. [94]:

$$ \mathrm{MR}(W)=\frac{\dot{V}{O}_2\left(16+5.164\times RER\right)}{60}, $$

where $\dot{V}{O}_2$ was the oxygen consumption (ml O_2_ min^− 1^) and $RER=\frac{\dot{V}\ {CO}_2}{\dot{V}\ {O}_2}$. Because we did not measure $\dot{V}{\mathrm{CO}}_2$ in one of the systems, we used RER = 0.80 which leads to smallest error in the calculation of energy expenditure [95].

### Statistical analysis

Statistical modeling was done in R using packages lme4 [96] and stats [97], and package emmeans [98] for post-hoc comparisons of estimated marginal means. Initial maximal models were built using ecologically relevant explanatory variables and their interactions, which follows the Regression with Empirical Variable Selection approach [99]. We also used second-order Akaike information criteria (AICc) for post-hoc model comparisons (MuMIn [100]). All results are presented as estimated marginal means ± SE compared pairwise with Tukey’s HSD test adjusted for multiple comparisons [98]. Statistical significance was accepted at *P* ≤ 0.050.

First, we compared life history traits of individuals from all experimental groups. Up to 32 day of life *m*_b_ of individuals was determined as the mass of whole litter divided by litter size. Between 45 and 90 days of life all hamsters were weighed approximately once a week (± three days). Analysis of life history traits was done in three steps: 1) analysis of *m*_b_ measured before separation of siblings (growth rate between birth and day 32); 2) analysis of *m*_b_ between days 45 and 90; 3) analysis of *m*_b_ just prior to transfer to SP. The first two analyses were done using linear mixed-effect modeling (LME; lme4 [96]) with type III Sums of Squares. The last analysis was done using general linear model (GLM; stats [97]).

In the model which tested the effect of litter order on offspring *m*_b_ up to 32 day of life, parental ID was included as a random factor to control for repeated measurements. The final model included litter order (first or third litter), offspring age, litter size, *m*_b_ of dam, and litter order × age and litter order × litter size interactions (Table 1). In analysis of offspring *m*_b_ between ~ 45 and ~ 90 day of life, animal ID was used as a random factor, and litter order, sex and age were used as fixed factors. The best model also included interactions of age × litter order and litter order × sex (Table 1). In the analysis that compared *m*_b_ of individuals acclimated to LP for 3 (groups II and III) and 5 months (groups I and IV) prior to transfer to SP, litter order, duration of LD acclimation, and sex were included as fixed factors and the final model included only main effects of these variables (Table 1).

To test the effect of litter order and duration of LP acclimation on offspring photoresponsiveness we used contingency tables with Pearson’s χ^2^ test (stats [97]). To do so we defined categories of torpor use and molting. Molting categories included white individuals if they molted after 16 weeks under SP or grey if they did not molt at all. A separate category included individuals that presented late response and molted later than after 20 weeks under SP. Torpor categories comprised individuals using or not using torpor within 16 weeks of acclimation to SP and individuals using torpor later than after 20 weeks in SP. Effects of litter order and duration of LP acclimation were tested separately. We tested also for the effect of interaction of the above factors on molting and torpor use using Cochran-Mantel-Haenszel test (stats [97]).

To assess the effect of litter order and duration of LP acclimation on *m*_b_ changes triggered by SP we used GLM (stats [97]). We calculated proportional change of *m*_b_ using last measurement taken in LP as the initial *m*_b_. Litter order, duration of LP acclimation, sex, categories of torpor use and molting were included as fixed factors and initial *m*_b_ as a covariate. The final model included all factors and interactions of sex × torpor use and sex × molting (Table 1).

Changes of *m*_b_ between 16th and 40th week of acclimation to SP in late responding individuals are presented descriptively because these individuals differed in winter traits and also in timing of their development.

To test weather polymorphism of winter phenotype among littermates was affected by litter order we used contingency tables with Pearson’s χ^2^ test. We classified litters as homogenous if all littermates presented the same phenotype and heterogeneous if littermates presented varied phenotypes.

To test for the effect of litter order and time spent in LP on BMR we used LME (lme4 [96]). We did three separate analyses: 1) to test for the effect of litter order on BMR in 3-months-old animals, 2) to test for the effect of litter order and duration of LP acclimation on changes in BMR triggered by photoperiod, and 3) to analyze changes in BMR in late responding individuals. All models included animal ID and trial as random factors that allowed to control for repeated measurements. Body mass was always included as covariate. The analysis of BMR in 3-months old animals included litter order and sex as fixed factors and their interaction (Table 4). To test for the effect of experimental group and development of winter traits on photoperiod-triggered changes in BMR we used litter order, duration of LP acclimation, photoperiod, sex, and winter traits (molting and torpor use) as fixed factors. The final model included fixed factors except torpor use and all interactions between duration of LP acclimation, photoperiod and molting and between litter order, photoperiod and molting (Table 4). In analysis of further changes of BMR in late responding individuals litter order, duration of LP acclimation, sex, molting and time of measurement (in LP, in SP and later in SP) were included as fixed factors. The final model included all main effects and interaction of litter order and time of measurement (Table 4).

## Data Availability

The datasets used and/or analysed during the current study are available from the corresponding author on reasonable request. Code will be available upon request to the authors.

## Abbreviations

BMR: Basal metabolic rate
LP: Long photoperiod
*m*_b_: Body mass
SP: Short photoperiod
